# Community engagement approaches and lessons learned: a case study of the PRECISE pregnancy cohort study in Kenya

**DOI:** 10.3389/fpubh.2025.1439150

**Published:** 2025-03-11

**Authors:** Onesmus Wanje, Angela Koech, Mai-Lei Woo Kinshella, Grace Mwashigadi, Alice Kombo, Grace Maitha, Nathan Barreh, Hiten D. Mistry, Marianne Vidler, Rachel Craik, Marie-Laure Volvert, Peter von Dadelszen, Marleen Temmerman, The PRECISE Network, Laura A. Magee, Joseph Akuze, Abdoulie Bah, Yorro Bah, Mwanajuma Bakari, Benjamin Barratt, Christine Baya, Kelvin Baya, Hannah Blencowe, Helena Boene, Jeff Bone, Carla Carrilho, Judith Cartwright, Rachel Craik, Rachel Craik, Umberto D’Alessandro, Shilla Dama, Brahima Diallo, Modou F.S. Ndure, Veronique Filippi, Gibril Gabbidon, Ursula Gazeley, Lawrence Gibba, Yahaya Idris, Hawanatu Jah, Consolata Juma, Sharon Konde, Fatoumata Kongira, Sanjeev Krishna, Joy Lawn, Jing (Larry) Li, Salesio Macuacua, Sonia Maculuve, Liberty Makacha, Inacio Mandomando, Melisa Martinez-Alvarez, Thomas Mendy, Hiten Mistry, Reason Mlambo Lucilla Poston, Sophie Moore, Moses Mukhanya, Peris Musitia, Joseph Mutunga, Emily Mwadime, Isaac Mwaniki, Baboucarr Njie, Alison Noble, Marvin Ochieng, Patricia Okiro, Geoffrey Omuse, Aris Papageorghiou, Kelly Pickerill, Andrew Prentice, Lazaro Quimice, Anna Roca, Donna Russell, Tatiana Salisbury, Ash Sandhu, Abdul Sesay, Esperança Sevene, Matt Silver, Sambou Suso, Antony Tangai, Prestige Tatenda Makanga, Corssino Tchavana, Fatima Touray, Rachel Tribe, Domena Tu, Anifa Vala, Marie-Laure Volvert, Guy Whitley, Irene Yaa

**Affiliations:** ^1^Centre of Excellence for Women and Child Health, Aga Khan University, Nairobi, Kenya; ^2^Department of Obstetrics and Gynaecology, Aga Khan University, Nairobi, Kenya; ^3^Department of Obstetrics and Gynaecology, University of British Columbia, Vancouver, BC, Canada; ^4^Department of Women and Children’s Health, King’s College London, London, United Kingdom; ^5^Department of Population Health Sciences, College of Life Sciences, University of Leicester, Leicester, United Kingdom; ^6^Faculty of Medicine and Health Sciences, Ghent University, Ghent, Belgium

**Keywords:** community engagement, community participation, cohort studies, pregnancy, Kenya, lessons learnt

## Abstract

Community engagement (CE) has been recommended as an important ethical consideration for health research to enhance informed consent and exchange knowledge between researchers and community members. The purpose of this paper is to describe how CE was developed and delivered for the PRECISE prospective pregnancy cohort study in Kenya. PRECISE enrolled pregnant women in antenatal care, followed them up to the postpartum period, and collected data and biological samples to enable the study of placental disorders in sub-Saharan Africa. Initially CE was aimed at informing the community about the study, establishing community-wide acceptance of the research and addressing concerns about biological sample collection to facilitate participation in the study. CE later evolved to be a platform for mutual learning aiming to deepen the community’s understanding of research principles and informed consent and providing a feedback loop to researchers. We engaged diverse stakeholders including health workers and managers, local administrators, religious and traditional leaders, older women, pregnant women, non-pregnant women and men. We utilized a variety of CE approaches and tools adapting to the specific contextual factors at the study sites. Achievements included widespread understanding of informed consent and research principles, clarification of misconceptions, and dispelling of fears regarding biological sample collection. The relationship with the community was strengthened evidenced by frequent inquiries and active participation in CE activities and the research study. For effective CE, we recommend involvement of community members in the CE team and continuous and adaptive CE throughout the study period.

## Introduction

Community engagement (CE) is important in maternal health (1), with the understanding that decisions around antenatal, delivery, and postnatal care are usually taken by women and their relatives as members of broader communities (2). Community engagement (CE) is a dynamic process that entails collaborative work with and through a varied groups of people to address issues impacting their well-being (3). In this article, the term “community” has been adapted to include various groups, including residents of the settlements where health research is conducted, potential study participants, all other residents nearby as well as stakeholders from outside the area (e.g., healthcare workers, women group leaders). In Kenya, household decision-makers such as husbands, mothers-in-law, and community leaders heavily influence women’s decisions around healthcare seeking and engagement in health research (4, 5). CE has been shown to be effective in improving health behavior and access, health literacy and self-efficacy, health outcomes, perceived social support and public health planning, even among disadvantaged groups who face barriers in accessing health services (6, 7). It can serve as a vital component in health interventions aimed at promoting health, extending beyond mere health promotion. CE embodies a mutually educative process involving researchers and participating communities.

CE is a critical aspect of medical research as per the Council for International Organization of Medical Sciences (CIOMS) (6). Ethicists recommend it, some research funders require it, and ethics guidelines advocate for it (8). It has been recommended as an important consideration for health research in Africa, particularly research using biobanks (7) in order to enhance informed consent and ethical research (9), gain knowledge of relevant community perspectives, beliefs and practices, and knowledge sharing (8). When applied effectively in research, CE can result in wide involvement of the community in research including participation in research studies (10). Inadequate CE, however, could lead to mistrust and undermine the use of informed consent leading to low participation in research studies (11). Although CE is increasingly valued as part of ethical engagement with research studies, there is little guidance on how to develop and deliver meaningful engagement (7, 10, 12), particularly in Africa. Further, the diverse settings where research is conducted call for approaches to CE that consider the unique contextual factors at each setting (13).

CE embodies a mutually educative process involving researchers and participating communities. CE has been described as a continuum with varying degrees of community and researcher involvement and activity (14). The Continuum of Community Engagement in Research (CEnR) framework describes these degrees ranging from ‘no community involvement’ to ‘community informed’, ‘community consultation’, ‘community participation’, and all the way to ‘community led’. Researchers may implement CE at various degrees in the continuum approaches as appropriate to their context, goals and priorities (14).

The PRECISE (PREgnancy Care Integrating Translational Science, Everywhere) Network established a multi-country prospective pregnancy cohort to study placental disorders in sub-Saharan Africa (15). PRECISE was implemented in Kenya, The Gambia and Mozambique and included four study visits until postpartum with detailed collection of social, clinical, environmental, and demographic data, as well as biological samples (maternal blood, urine, vaginal swabs, cord blood, placenta). Biological samples were stored in a biorepository to enable future research to identify biological markers and mechanisms leading to placental disorders and other pregnancy complications (16). CE was identified early during the project set-up as a pre-requisite for community acceptance and involvement in research, particularly for addressing concerns about biological sample collection (16). It was also used as an avenue to foster mutual learning between researchers and community members (17). Initially CE in PRECISE was aimed at informing the community about the study objectives and activities, establishing community-wide acceptance of the research and addressing concerns about biological sample collection to facilitate participation in the study. CE later evolved to be a platform for mutual learning aiming to deepen the community’s understanding of research principles and informed consent and providing a feedback loop to researchers.

The purpose of this paper is to describe how we developed and delivered CE initiatives for a pregnancy cohort study in Kaloleni and Rabai in Kilifi, Kenya. In discussing the CE approach and the level of community involvement, we have applied the Community Engagement Continuum framework (14).

## PRECISE study sites in Kenya

**Figure 1:**
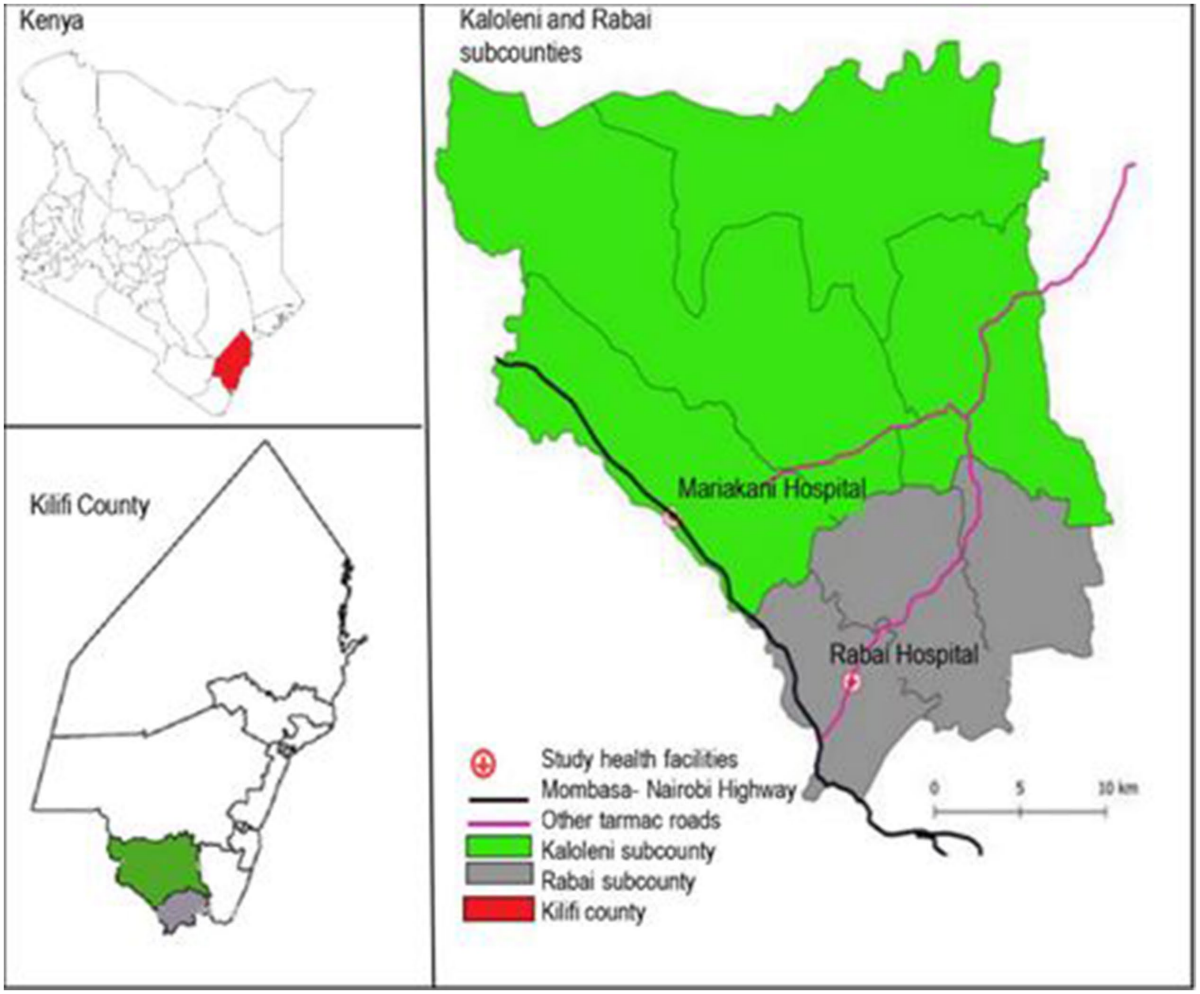
Map of Kaloleni and Rabai sub-counties showing the study sites for the PRECISE study in Kenya (46).

The PRECISE study in Kenya was conducted by the Aga Khan University’s (AKU) Center of Excellence in Women and Child Health - Kenya, in collaboration with the Kilifi County Department of Health (15). The research activities were centered at Rabai Health Centre (now Rabai Sub-county Hospital) and Mariakani Sub-county Hospital, both public hospitals in Kilifi County that are managed by the Department of Health. Rabai Hospital is in Rabai Sub-county and serves a predominantly rural population. Mariakani Hospital is located in Mariakani town and serves its urban and peri-urban population but also receives referrals from other facilities in the wider Kaloleni Sub-county (Figure 1). The two sub-counties cover an area of about 910 km^2^ and have a combined population of 314,495, 52% of whom are female (18). Approximately 40% of the population are Christian, 40% are Muslim and 8% are traditionalists (19). Approximately 70% of the population live below the poverty line (20). Pregnancy care-seeking behavior has improved in recent years, being as high as 99% of women having at least one contact with a skilled provider in pregnancy and 85% having attendance by skilled personnel at birth (21). According to the Kilifi County Fiscal Strategy Paper of 2021, the overall literacy rate in Kilifi County is 68%, with the male population comprising 51% and the female population comprising 49% (22).

Various national and international aid as well as development organizations have been active in the area, implementing several health and development projects; however, clinical research studies have been relatively few. In 2017, AKU established the Kaloleni Rabai Community Health and Demographic Surveillance System that collects routine health related data periodically from households (20). The PRECISE study was established in 2018 and participant enrolment in the area started in July and November 2019 in Rabai and Mariakani, respectively. To date, PRECISE is the largest clinical research study focusing on pregnancy health in the area.

## Developing the community engagement strategy

Our CE strategy was developed with reference to previous studies that focused on creating culturally appropriate CE strategies in similar low-resource settings, including those conducted in Kilifi (23, 24). These studies highlighted the importance of CE in research and building community capacity through locally relevant approaches. While we were influenced by the previous work that partnered with existing community members, we also aimed to innovate and adapt our approach to the specific needs of our study context. While KEMRI’s CE strategy utilized KEMRI community representatives (KCRs) (23), our approach differed by engaging Community Health Volunteers (CHVs). The process of recruiting and maintaining KCRs is often expensive and time-consuming. In contrast, CHVs were already established within the community and were actively supporting the Ministry of Health (MOH) as well as other research initiatives like the surveillance project (20). This allowed us to engage with them immediately without requiring the establishment of a new structure. Moreover, like KCRs, CHVs are members of the community and therefore, able to represent community perspectives.

### Consultations

To refine our CE activities, key messages, and stakeholder mapping, our research team engaged in consultations with other organizations conducting health research in the region to understand their approaches to CE. Moreover, we consulted healthcare workers and managers at the two study health facilities, Rabai and Mariakani hospitals and the health management teams in Kaloleni and Rabai Sub-Counties. We also held internal consultations within AKU between the PRECISE staff and other project teams, and PRECISE staff joined community meetings organized by other projects to improve understanding of the local context.

### Establishing the community engagement team

The project recruited a social scientist to coordinate and implement the project’s CE and community research activities. Two research assistants were later hired by the project to support these activities. Proficiency in the local language and knowledge of the area’s geography and culture were key factors considered in the selection of staff. The research assistants were then trained on research principles and effective communication approaches with the bulk of the training delivered by the CE lead / social scientist. Other staff members of PRECISE, including investigators, the study coordinator, and the laboratory lead, regularly joined CE activities.

By the end of PRECISE’s recruitment phase (February 2022), the CE team had eight members, with a majority from the local Mijikenda ethnic group who spoke the local language and were familiar with socio-cultural contexts at the study sites. Involvement of researchers who spoke the local language encouraged open dialog with communities, which supported implementation of the research in a culturally sensitive manner. Continued dialog with research assistants from the local community throughout the CE processes was in itself a platform for ‘community consultation’ and they gave various views on the research processes and CE approaches on behalf of the community.

### Developing key messages

Messages were informed by CE goals and were developed in discussions within the PRECISE Kenya staff as well as in collaboration with the global PRECISE Network members. We free-listed all possible messages, then prioritized them to align with the CE goals.

Initially, the content covered in our engagement sessions was very extensive, prompting us to reduce the scope and focus on messaging introducing research principles, informed consent and explaining the study objectives and procedures including biological samples collection. Because research was novel in this community, creating awareness on what research was and its principles was foundational. Messages were developed around these key topics (Table 1) but were designed to be flexible to adapt to issues highlighted in preceding sessions.

**Table 1:** Key messages.

Key Topics	Discussion points
Introduction to research and research principles	What research is and how it differs from routine health care & development projects? Research ethics. Benefits of health research
Informed consent	The process of informed consent Voluntary participation and freedom to withdraw from research. Access to standard health care services not contingent on agreeing to participate in research
Biological sample collection	Sample types and methods of collection. Purpose and value biological samples
Addressing misconceptions	Addressing myths and misconceptions about PRECISE study Responding to questions and concerns about PRECISE study raised by the community members.

### Mapping the geographical scope of community engagement

Before initiating the engagement activities, we outlined the community health units and villages where we would conduct our engagement activities. The first phase of engagement included conducting community entry meetings across most community health units (CHU) and villages in the two sub-counties. In Kenya, a CHU is a health service delivery structure within a defined geographical area covering a population of approximately 5,000 people (25). Later, we refined our focus by reviewing antenatal care registers at Mariakani and Rabai Hospitals to identify the CHUs and villages where most of the pregnant mothers in PRECISE lived. Rather than across the entire sub-countries, we narrowed the geographical scope down to 8 CHUs in Mariakani and 7 CHUs in Rabai where most women attending antenatal care at the two hospitals resided. Narrowing down the geographical scope was essential for ensuring the relevance of our engagement activities by directing our efforts toward communities where it was necessary and likely to yield meaningful results, particularly for pregnant mothers accessing antenatal care at our two-study site.

### Mapping the stakeholders and engaging gatekeepers

**Figure 2:**
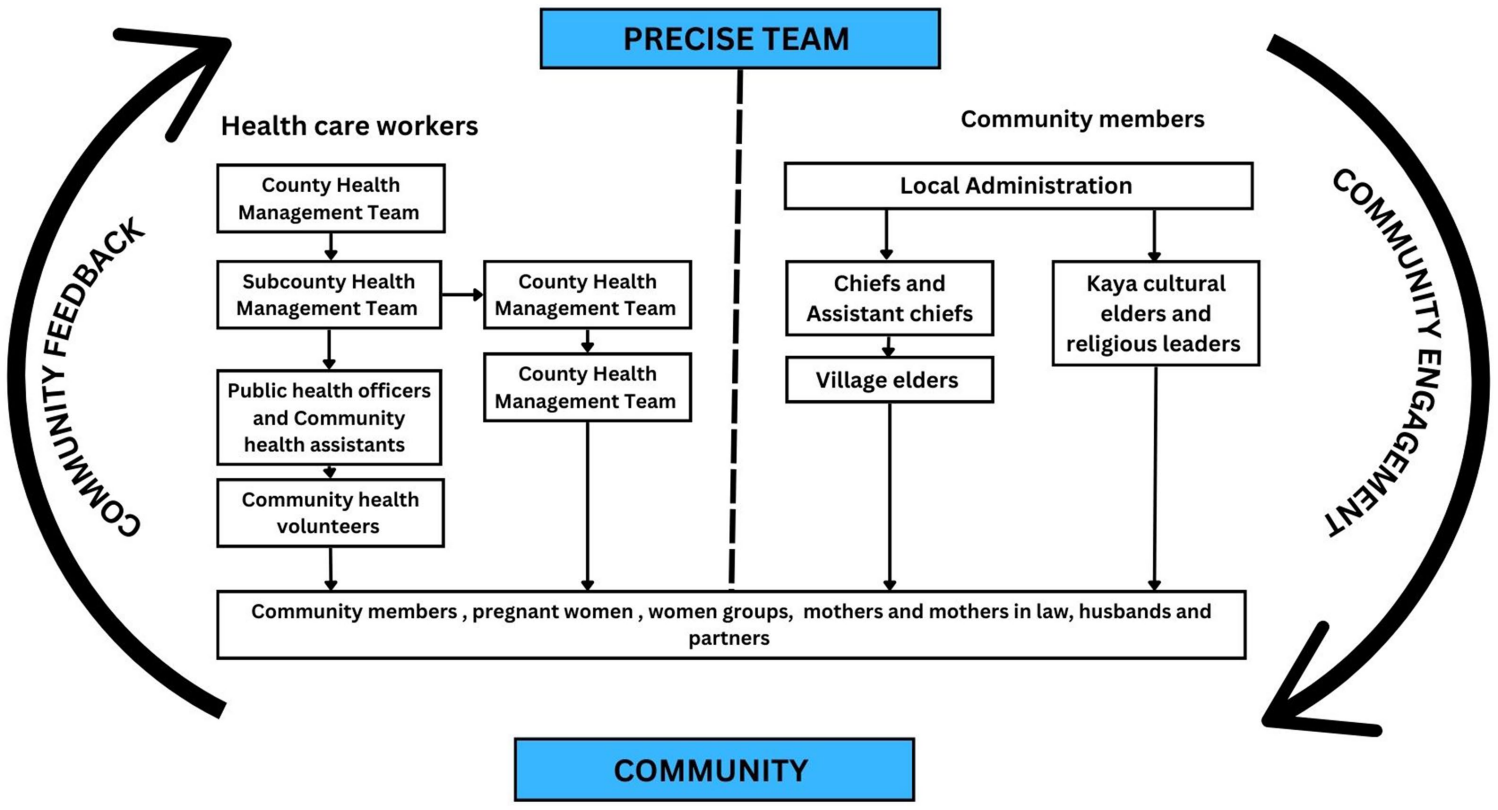
Pathway of community engagement and target groups.

First, we freely listed potential stakeholders in the two study areas informed by the prior consultations. We reviewed the list to determine the most significant stakeholders for the study based on individuals with an interest in the research project and those from whom we required their support. We later developed a pathway of stakeholder engagement to reach the specific target groups identified (Figure 2). This targeted approach to identify and work with key gatekeepers, such as area chiefs and religious leaders, was fundamental in building trust and creating spaces for meaningful dialog. Community gatekeepers appreciated their early involvement and felt that their roles in the community were respected by the researchers.

## Community engagement approaches

### Community engagement activities

CE methods included meetings with healthcare workers, meetings with community members and health talks at the two health facilities where the PRECISE study was conducted (Table 2).

**Table 2:** Community engagement meetings and participants reached.

Meeting category	Total number of meeting participants
County Health Management Team and other county level health managers	35
Sub-county Health Management Teams	143
Health professionals	173
Women groups	199
Community Health Volunteers	1,374
Village elders	167
Kaya elders	28
Religious leaders	35
Husbands and male partners	195
Targeted pregnant and recently pregnant women	432
Mothers-in-law	25
Total	2,806

### Community engagement meetings with healthcare workers

We held meetings with healthcare workers at different levels of responsibility and cadres beginning with meetings with the county and sub-county health management teams (SCHMT) – the top leadership teams overseeing activities at the Department of Health and Sanitation Services at the county (Kilifi) and sub-county (Kaloleni and Rabai) levels, respectively. County meetings were held annually, and sub-country meetings were held biannually. Typical attendance was about 35 persons per meeting. At these meetings, we introduced the study and discussed research goals and approaches in detail. We then sought feedback and addressed concerns (Table 3). The management teams were keen to be involved in the planning and implementation of the CE activities. In subsequent meetings, they helped to identify the target groups and supported the gatekeeper engagement pathway (Figure 2).

**Table 3:** Concerns and questions raised about the study from community members, health workers and other stakeholders.

Why is research participation voluntary / optional yet it has good benefits to the mothers? Why cannot men participate in the study? If a mother agrees to participate in the study, does not it mean she will have two separate antenatal care clinics? If a participant is confirmed to be hypertensive will she receive any treatment or not? If a research participant faces a complication at delivery and is referred to another hospital, will the study pay her bills? After giving out my samples as a participant, what benefits will I get? After getting the outcomes and the results of PRECISE Study, will Aga Khan University give us the results? Why is blood collected in three different tubes? What would happen if the samples collected are found to have problems? Will the participant be informed of the results immediately? Where will the placenta samples be taken to? From the placenta, are you taking blood or the section of the flesh? Is it possible to have participants witness the placenta collection procedure? Can a mother get an infection from vaginal swab collection?

We then convened meetings with health facility staff with attendance drawn largely from nurses working at the maternity ward and antenatal clinics, as well as staff from other sections such as the laboratory and other inpatient wards. These meetings had an average attendance of 40 persons and were held twice a year. At these meetings, we described the research study and its goals, the differences between routine care and research, and the importance of voluntary participation. Discussions with the staff informed the design of participant flows at the antenatal clinics and maternity wards. Later, health facility were formally engaged in the research performing tasks such as participant screening, information giving, clinical data collection and selected study procedures, a form of ‘community participation’ in research. Hospital nurses were often approached by pregnant women seeking a second opinion about the study as they were familiar to them and more trusted.

We had similar engagements with public health officers and community health assistants where we introduced the study and sought guidance on how best to engage with the community. Public health officers and community health assistants then joined the PRECISE CE team in further engagements with CHVs. In Kenya, CHVs (also known as community health promoters) are trained members of the community who work as a link between the community and formal health facilities. They play a crucial role in addressing health inequalities by serving as a link between the community and formal health facilities and bringing essential health services closer to the people, especially in rural and underserved areas (25). These meetings with CHVs aimed to strengthen relationships between them and the researchers, while also equipping CHVs to respond to questions from women and other community members about PRECISE, given their close contact with community members. CHV meetings were held twice a year and had an average attendance of 35 persons. CHV meetings were rotational to cover all the community units in the study areas. We provided detailed information about the study and shared updates on the study progress. In turn, CHVs gave feedback from community members on any emerging issues and concerns and jointly discussed solutions to these concerns. CHVs then participated in subsequent meetings with other community members helping to facilitate sessions and deliver some key messages. This ‘community participation’ by CHVs built trust and strengthened relationships.

### Engagement with pregnant women through health talks

Daily health talks delivered by ANC nurses in the mornings at ANC waiting areas are a routine part of the ANC visit covering various aspects of ANC, birth planning and knowledge of danger signs in pregnancy. Health talks are a widely used method of health education in health facilities worldwide. The PRECISE CE and clinical staff (nurses and other research assistants) joined these daily health talks to discuss the research study. The talks were delivered in the local language and visual aids such as posters were used. Participating pregnant women had the opportunity to ask questions during the talks or follow up with individual research staff they had been introduced to. The activities here were largely about informing the potential participants about the research study.

### Community engagement meetings with community members

The PRECISE CE team collaborated with public health officers to schedule community meetings and occasionally joined pre-existing “community dialogues” organized by community health teams of the Kilifi County Department of Health to engage communities in voicing their health concerns. Further CE meetings were organized in the community, engaging a total of 914 participants, including pregnant women (n = 432), members of women’s group (n = 199), participants from men’s groups (n = 195), mothers-in-law (n = 25), religious leaders (n = 35), and Kaya elders (n = 28). Kaya elders are respected members of the Mijikenda community who play a significant role in providing blessings, interpretation services, and championing unity within the Coast region of Kenya. The Kaya elders are essential figures in maintaining the spiritual and cultural identity of the Mijikenda community.

Public health officers mobilized meeting participants with the support of CHVs. These meetings were dialog-based and consisted of a welcoming and introduction by the public health official followed by a round of introductions. PRECISE CE staff introduced AKU as well as the PRECISE project and then discussed the key messages. This was followed by a question-and-answer session for community members to ask questions about the study and raise any concerns. A sample of frequently asked questions is provided in Table 3. A public health official made public health announcements before ending the meeting. Refreshments would be served, and attendance taken. Through these community meetings, the study was able to reach more community members with information about the study. We conducted an average of eight community meetings a month with different target community members. Typical attendance included 25–30 community members, 3 public health officials and 3 CHVs at these meetings.

Meetings with men were also held in the two study sites to inform them of the study, with around 195 husbands reached. Husbands or male partners have a lot of decision-making power within the household (4, 5). CE activities allowed for husbands and partners to be informed early about the study giving them the opportunity to discuss the research study with their wives prior to their antenatal care visits. We noted that women who were informed about the study before their health facility visit seemed more empowered to provide consent without additional consultation with their spouses.

## Community engagement tools and materials

### Visual aids

**Figure 3:**
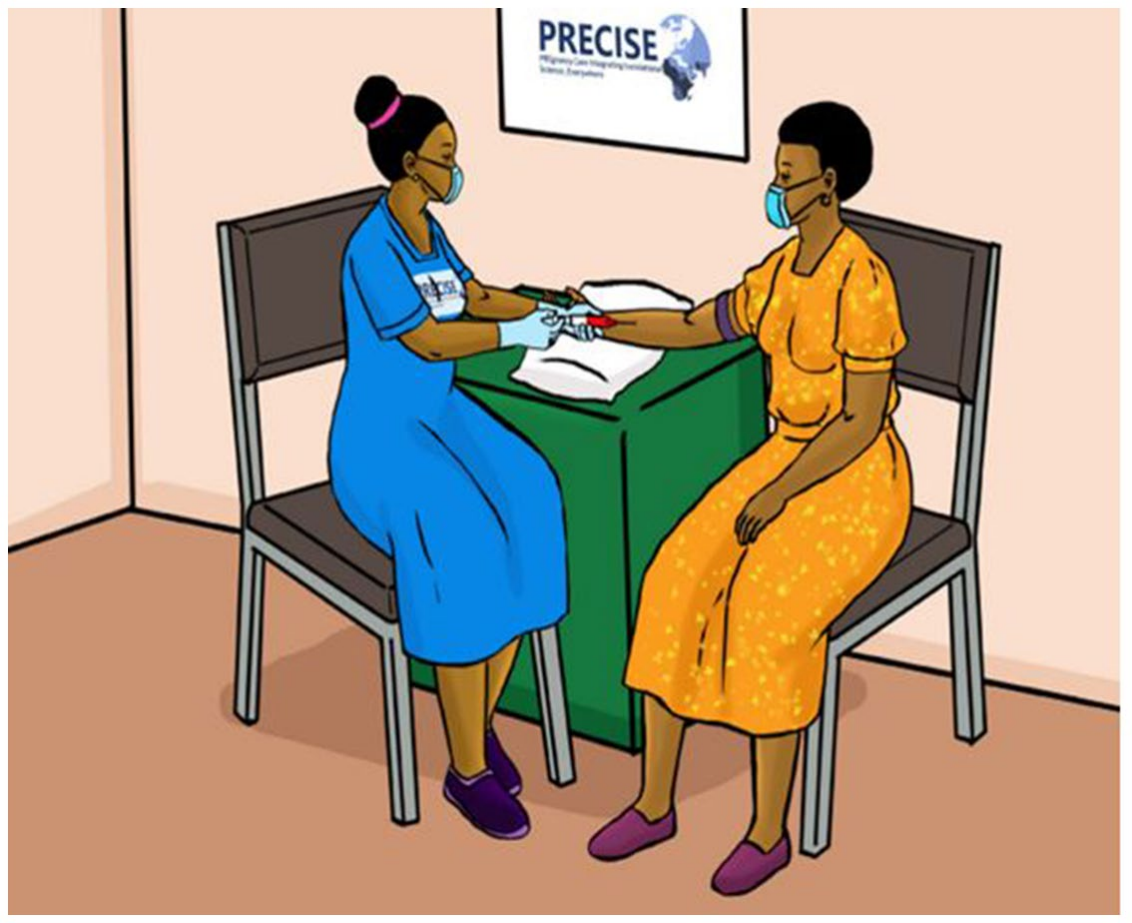
Pictogram of PRECISE lab staff collecting blood from a mother’s vein.

Visual aids utilized included PowerPoint slides, pictograms, posters, and flyers. Pictograms (for example, Figure 3) illustrated study procedures to visually complement verbal information. Visual aids can significantly improve health literacy among low literacy populations (26) and we found the pictograms to be particularly useful as they enhanced understanding of the messages around the study procedures. Moreover, the team designed Swahili posters and flyers, written in simple language, to inform pregnant women and other community members about the study. Flyers were distributed at CE meetings and at health facilities, while posters were displayed at the study hospitals’ notice boards. Flyers are a versatile and effective means of engaging communities in various health research projects, and they have been widely applied in various fields of research (27, 28).

### Interactive methods in community engagement activities

We utilized various interactive methods to encourage active participation and comprehension of the key messages. These methods included role-playing scenarios, discussing case studies, and a sing-along song about the value of research (Box 1). We also showed actual research items such as test tubes and syringes to demonstrate the volume of blood collected in the research study and encouraging participants to handle these items. These tools were very effective in triggering dialog and enhancing understanding and retention of messages, in the contextual background of low literacy levels. Interactive methods have found wide application in health research (29–31).

**Box 1 Examples of interactive methods**

- Tuskegee case study – Discussion of the Tuskegee study (32) conducted in the United States from 1932 to 1972 where hundreds of men with syphilis were left untreated, even after discovery of penicillin, and without their informed consent to observe the natural progression of the disease. The case study was used to discuss principles of research ethics including informed consent, respect for participants, beneficence, and the need to minimize harm to research participants.
- Malaria prophylaxis in pregnancy case study – Malaria prophylaxis during pregnancy was discussed as an example of a future benefit of health research. Previous studies demonstrated the effectiveness of intermittent preventive treatment with antimalarial drugs (33) on reducing the incidence of malaria during pregnancy. These studies led to the wide implementation of the intervention for all pregnant women in malaria-endemic areas. Pregnant women resonated with this case study as they were familiar with intermittent preventive treatment from their antenatal care visits.
- Participatory bus scenario – Attendees were invited to board an imaginary bus parked outside the meeting room/ venue. Minimal information was provided about the bus or the intended destination. The willingness of attendees to board the bus was used to check understanding about informed consent. Attendees were encouraged to ask extensive questions before agreeing to participate in any research study.
- “*Utafiti ni nini*?” (What is research?) song - Composed in Swahili by the PRECISE CE team and sang to the tune of a famous local song, the song echoed the key messages about the process and benefits of health research (full lyrics in Table 4).

## The role of community feedback

‘Community consultation’ is a level of CE where advice and guidance is taken from the community and utilized to inform the research activities. The CE strategy was evaluated with continuous reflection through discussions within the PRECISE Kenya team and feedback received would be applied in subsequent CE meetings. Community feedback also informed the study processes and procedures, especially the administration of informed consent (see Figure 2). Frequently asked questions were relayed to the research assistants to better prepare them for informed consent sessions. The PRECISE CE team met to debrief/evaluate after every meeting on what went well, what to improve, and any difficulties experienced; CE activity reports were then completed by the facilitators. Information recorded included date and venue of the meeting, number of participants overall as well as by target group, issues discussed, community feedback, questions, and concerns. The community liaison officer monitored CE activity reports and reported to the wider PRECISE Kenya team at regular research meetings that were held every 2 weeks on any emerging issues and concerns from the community. The CE strategy was evaluated with continuous reflection through discussions within the PRECISE Kenya team and feedback received would be applied in subsequent CE meetings. Community feedback also informed the study processes and procedures, especially the administration of informed consent (see Figure 2). Frequently asked questions were relayed to the research assistants to better prepare them for informed consent sessions.

## Community engagement achievements

As explained below, the CE efforts appear to have increased awareness about informed consent and research, clarified misconceptions about PRECISE, addressed local concerns regarding biological sample collection, and fostered stronger relationships with community members and health workers.

### Increased awareness about informed consent and research

Earlier CE sessions confirmed little research experience in study communities, which impacted women’s capacity to provide informed consent. Initially, many women appeared to feel compelled to participate due to concerns about potential denial of services at health facilities if they declined participation in the study. CE played a key role addressing such beliefs by raising awareness about the research process and ethics, with a particular emphasis on informed consent and autonomy.

The rich discussions and questions asked on the subject demonstrated that community members gained understanding of the concept of consent especially involuntary participation. The study continued to experience a high acceptance of enrollment and continued attendance of subsequent study visits. The study proceeded without any interruptions.

### Clarifying misconceptions about PRECISE and addressing local fears regarding biological sample collection

Misconceptions elicited from community members and addressed during CE included concerns that PRECISE was collecting blood to sell and that the study collected “pints” of blood rather than the actual 16 mL of blood. Another concern was the belief that researchers were tampering with the placenta, which would influence future generations. This was likely fueled by a traditional belief that a child would become infertile if the umbilical cord stump touched its genitalia in the newborn period. Similar beliefs have been observed in other African cultural contexts (34). To address these concerns, detailed explanations on the blood volumes and placenta processing procedures were discussed in detail to dispel these fears and providing clarity to such misconceptions.

Initially, women expressed reservations about collection of study samples, particularly the placenta and cord blood. They held the belief that these samples should be disposed off without interference as a sign of respect for their newborns. However, they were subsequently guided through the study procedure, with detailed explanations of what would be done to the samples and their potential value in understanding pregnancy and newborn health. After these discussions, participants in the meetings understood the sample collection process and expressed gratitude for being well-informed.

Similarly, the Kaya elders, who serve as custodians of the Mijikenda culture and beliefs, initially they had concerns regarding the collection of placenta samples. However, after receiving detailed descriptions of the placenta sample collection and analysis process, they granted their cultural approval.

### Strengthening relationships

We succeeded in establishing a strong relationship with community members in the study area. An unplanned break to CE activities due to staff turnover and COVID-19 prompted numerous inquiries about the status of the study especially whether it was ongoing or halted. Study participants would approach research staff ‘on the street’ with questions about the study or health care in general. Upon discharge from maternity post-delivery, some participants would drop by the research offices just to say ‘hello’ and update us on their pregnancy outcome.

There was a good sense of co-ownership between the PRECISE study staff and the health workers. Healthcare workers would provide real-time feedback to the study team upon hearing any negative information about the study from the community. This collaborative ownership was also apparent during community meetings, where healthcare workers would actively participate and engage with the study team throughout the sessions, frequently responding to questions together.

Engagement with community gate keepers, including religious leaders, chiefs, Kaya elders and women group leaders was appreciated and helped to open channels of dialog and build trust of the study by the community members. These engagements helped to build local interest and engagement in the research. Most of the Kaya and religious leaders felt honored and respected by being officially engaged in health research for the first time. Area chiefs also appreciated PRECISE for recognizing their crucial role in the community. In rural African contexts, researchers need to build trust with gatekeepers such as chiefs and elders to involve participants in culturally appropriate ways as cultural values and traditions heavily influence the research process (27).

## Facilitators, barriers and recommendations for future engagement

### Facilitators

Like the key CE components previously reported (35), facilitators of meaningful engagement in our PRECISE study included a dedicated CE team recruited from local community who are familiar with the local context. By residing in study communities, it helped to build strong relationships between community members and the project and to overcome traditional barriers to research engagement by considering the social and historical elements of the study area. These are key contextual factors within the CE continuum. This led to community members trusting the project enough to ask questions and raise misconceptions such as collecting blood to sell, which could then be addressed rapidly. Additionally, use of interactive methods and visual aids helped to promote participation and deliver messages in a population with low literacy. The integration of the study within the existing health system structures was also a key facilitator. Husbands and partners have a lot of decision-making power within the household (4). CE activities have been found in other studies to promote early engagement of husbands and partners, promote maternal health and educate on potential obstetric complications (36–40), which concurs with what we observed in our CE activities.

The research team received additional funding to continue the follow-up of the PRECISE pregnancy cohort. The PRECISE-Dyad study (41) enrolled pregnant women and babies from PRECISE following them up to 3 years post-delivery. As a result, CE activities in PRECISE continued beyond the PRECISE recruitment period with seamless and gradual transition to the PRECISE-Dyad recruitment and follow-up period. This enabled the same CE team to continue their work and to build upon successful approaches adapting them for the cohort extension.

### Barriers

Barriers to our CE program included staff turnover, the poor conditions of roads especially during the rainy season and the COVID-19 pandemic which impacted on social interactions. Staff turnover disrupted the continuity of CE efforts, new staff members often required time to build relationships and gain the trust of community members, which impeded the progress of ongoing CE initiatives. The poor road conditions hindered the mobility of the CE staff, making it challenging to reach remote communities resulting in uneven participation and coverage. In-person meetings were halted between March 2020 and October 2020 during the pandemic to control spread of the infection (42). Meetings gradually resumed in late 2020 but several adjustments had to be made regarding meeting venues, number of participants allowed, and requirements to provide personal protective equipment at meetings to comply with local and national regulations. Other studies have reported similar barriers and adaptations to CE due to the COVID-19 pandemic (43).

### Recommendations

CE is a contextual activity, while our study’s experiences are unique to our area, the lessons learned can be valuable for similar rural African settings. Drawing from our experiences, we recommend the following specific strategies (Table 4).

**Table 4:** Research song.

**Soloist:** Utafiti ni nini? X2 (What is research) **All:** Uchunguzi wa kina x2 (In-depth evaluation) **Soloist:** Ukiwa na maswali x2 (If you have questions) **All:** Fanya utafiti x2 (Conduct research) **Soloist:** Kwa ulezi wa mimba x2 (For pregnancy care) **All:** Fanya utafiti x2 (Conduct research) **Soloist:** Kwa afya ya jamii (For the health of the community) **All:** Fanya utafiti x2 (Conduct research) **Soloist:** Mwisho wa utafiti? X2 (At the end of research) **All:** Faida kwa jamii x2 (Benefits to the community)

CE should be a continuous process with the community throughout the life of a study and not simply introducing a research study to local populations (44). It is also important to continue CE after the end of the project to disseminate the key outputs and funders should consider the importance of post-project CE when funding research projects. CE provides a forum for interaction and dialog especially important for addressing potential misunderstandings around the collection and storage of biological samples (45). Consequently, continuous dialog is essential for research, in populations with socio-economic deprivation and low literacy levels (44).

Secondly, CE staff should be familiar with local contexts and be engaged for the lifespan of the study if possible. Community members develop bonds with CE staff they regularly interact with. This builds trust and allows open dialog, particularly with sensitive topics like collection of biological samples for research. Partnerships with community representatives, such as CHVs, can help to channel community feedback and questions to the study staff. Practical lessons on management of community meetings are described in Box 2.

**Box 2 Practical lessons learned for management of community meetings**

- **Meeting location**: Moving meetings to community settings close to target audience promoted more participation compared to reimbursing transport costs to a central location.
- **Number of participants per meeting**: A group of around 25 people was optimal. Larger groups were easily distracted.
- **Timing of CE meetings**: Late morning was an optimal time for engaging mothers in these communities as it was after they had completed household chores and before time for preparing lunch for their families.
- **Meeting duration:** Meetings with mothers that lasted more than an hour were less effective as the women often brought their small children to the meetings.
- **Time allocation**: Facilitators should ensure longer time allocation for in-depth discussion of key messages within meetings.
- **Other meeting facilitators**: Providing milk for infants and young children helped to calm them during meetings and providing the adults with refreshments helped create a more comfortable and welcoming environment.

## Conclusion

CE played a crucial role in integrating the PRECISE study into the study communities. Our CE strategy went beyond improving research literacy and promoting research participation. Regular interaction and dialog between the research project and the catchment communities cultivated trust and established an environment where community members felt empowered to pose questions, provide feedback and in some cases participate in the research processes. Our experiences demonstrate CE moving along the continuum from ‘community informed’ about the research to ‘community participation’ in the research.

## Data Availability

The original contributions presented in the study are included in the article/supplementary material, further inquiries can be directed to the corresponding author.

## Group members of the PRECISE Network

Patricia Okiro, Geoffrey Omuse, Consolata Juma, Joseph Mutunga, Moses Mukhanya, Isaac Mwaniki, Marvin Ochieng, Emily Mwadime, Shilla Dama, Irene Yaa, Sharon Konde, Mwanajuma Bakari, Peris Musitia, Antony Tangai, Kelvin Baya, Christine Baya, Umberto D’Alessandro, Anna Roca, Hawanatu Jah, Andrew Prentice, Melisa Martinez-Alvarez, Brahima Diallo, Abdul Sesay, Sambou Suso, Yahaya Idris, Baboucarr Njie, Fatima Touray, Fatoumata Kongira, Modou F.S. Ndure, Gibril Gabbidon, Lawrence Gibba, Abdoulie Bah,Yorro Bah. Esperança Sevene, Corssino Tchavana, Salesio Macuacua, Anifa Vala, Helena Boene, Lazaro Quimice, Sonia Maculuve, Inacio Mandomando, Laura A. Magee, Rachel Craik, Marie-Laure Volvert, Hiten Mistry, Thomas Mendy, Donna Russell, Prestige Tatenda Makanga, Liberty Makacha, Reason Mlambo Lucilla Poston, Rachel Tribe, Sophie Moore, Tatiana Salisbury, Aris Papageorghiou, Alison Noble, Rachel Craik, Hannah Blencowe, Veronique Filippi, Joy Lawn, Matt Silver, Joseph Akuze, Ursula Gazeley, Judith Cartwright, Guy Whitley, Sanjeev Krishna, Jing (Larry) Li, Jeff Bone, Domena Tu, Ash Sandhu, Kelly Pickerill, Carla Carrilho, and Benjamin Barratt.
